# Increased *PTEN gene* expression in patients with endometrial carcinoma from areas of high risk depleted uranium exposure

**DOI:** 10.1186/s13104-019-4756-4

**Published:** 2019-10-29

**Authors:** Alaa Salah Jumaah, Hawraa Sahib Al-Haddad, Liwaa Hussein Mahdi, Emad Hatem, Asaad Abdul Hamza Al-Janabi, Katherine McAllister, Akeel Abed Yasseen

**Affiliations:** 1 Department of Pathology and Forensic Medicine, Faculty of Medicine, University of Kufa, Kufa, P.O. Box 21, Iraq; 2 Al-Furat Al-Awsat Hospital, Kufa, Najaf Governorate, Iraq; 3 Al-Diwaniya Gynecology and Pediatric Hospital, Al-Qadisiya Governorate, Iraq; 4 Centre for Cancer Research and Cell Biology, Queens University Belfast, Belfast, UK

**Keywords:** Depleted uranium, *PTEN gene*, Endometrial carcinoma

## Abstract

**Objective:**

Investigate *PTEN* gene expression and tumor aggressiveness in endometrial carcinoma specimens from patients living in either areas of depleted uranium [DU] pollution or unpolluted regions to determine any evidence for the effect of war pollution on the rising trends of cancer incidence in Iraq.

**Results:**

Tumor *PTEN* gene expression was significantly increased in patients living in the areas of high risk DU exposure, in comparison to patient tumors from low risk areas [P = 0.001]. The age distribution between the potentially DU exposed (55.09 ± 1.24) and unexposed subjects 56.38 ± 1.18) was not significant [P = 0.45]. Endometrial carcinoma aggressiveness was equivalent in both subject groups, with no significant differences in either tumour grade and [P = 0.286] stage distribution [P = 0.98]. Finally, there were no significant differences between the potentially exposed and unexposed subjects with regard to cervical [P = 0.532] or to ovarian involvement [P = 0.518]. The results linked environmental war pollutants [DU] to alterations in PTEN gene expression in endometrial carcinoma. Furthermore, this finding may explain the overall increasing cancer trends observed in Iraq. Strategies should be considered for the therapeutic targeting of cancers with elevated PTEN gene expression to improve patient outlook.

## Introduction

Endometrial carcinoma is the most common gynecological malignancy in the United States, Japan and other developing countries [1, 2]. Globally it comprises about 3% of all adult female malignancies [3]. There are no reliable studies available to determine incidence of this type of cancer amongst Iraqi women. However, over 380,000 new cases worldwide were diagnosed in 2018 alone [4]. This stresses the urgent need for scientists to tackle such a worldwide problem. There are also many predisposing or risk factors which are believed to play a vital role in the initiation of endometrial carcinoma. These factors may include: diabetes mellitus, hypertension, hormone replacement therapy, obesity and late menopause [4]. Many cases of endometrial carcinoma are sporadic and about 5% have a hereditary predisposition [5]. Endometrial carcinoma is a typical cancer, resulting from numerous genetic errors and alterations. Such alterations are probably caused by faulty repair of DNA damage causing the accumulation of genetic errors.

The most common genetic alteration (30–80%) in endometrial carcinoma [6] occurs in the PTEN gene [7]. PTEN is a tumor suppressor gene located on chromosome 10q23. The protein is involved in different cellular functions such as migration and proliferation [6, 8]. PTEN inactivation is brought about by mutations causing loss of expression and to a lesser extent by loss of heterozygosity [9, 10]. The PTEN protein plays a vital role in controlling the PI3K/AKT pathway by phosphorylating PIP3 at the cell membrane. Loss of functional PTEN protein leads to uninterrupted production of tumourigenic PIP3. MTOR is the major effector of the PI3K/AKT pathway, promoting the G1 cell cycle phase and apoptosis-regulating protein interactions [11].

Recent studies have reported that overall cancer incidence has increased at least twofold amongst the Iraqi general population—especially in conflict areas involving Iraqi insurgents and occupation forces. According to the Iraqi Cancer registry, the incidence of cancer increased sharply both after the first and second Gulf war’s [12]. In 1991, the incidence of all cancer types in the Iraqi population was reported to be around 31.05 cases per 100.000 people [12]. However, figures have sharply risen after the war in 2003 to reach a peak of 61.63 cases per 100.000 [12]. Yasseen and co-workers have linked this cancer risk to exposure to DU—a toxic heavy metal with radioactive properties deployed in weaponry [13, 14]. Unfortunately, environmental DU may increase the risk of carcinogenesis in the inhabitants of these polluted war zone areas. More specifically in the female population—DU may impact the pathogenesis of endometrial tumours. We investigated this possibility by quantifying PTEN gene expression profiles and tumor aggressiveness in endometrial carcinoma patients living in either a DU polluted or unpolluted environment.

## Main body of text

### Materials and methods

#### Study design and population

The study was carried out at the Department of Pathology and Forensic Medicine, Faculty of Medicine, University of Kufa for 1 year from October 2006 to October 2007. All cases were collected from major hospitals and private laboratories in the middle and south of Iraq. Study ethical approval was obtained from the local Ethics Committee of the University of Kufa, Faculty of Medicine and written consent obtained from each patient. The patients were females, age matched, Arab descend and obtained from the same geographical areas to exclude or minimize the effect of any confounder variables (e.g. age, gender, geographical distribution, ethnicity) on the final outcome. The residual confounder was not an issue as the present work considered all the possible confounding factors during the work, adjusted and controlled accordingly. The cross sectional observational study included 43 cases of endometrial carcinoma. Twenty-one cases came from a conflict region involving DU military activities. A further 22 samples were obtained from endometrial carcinoma patients living in a peaceful area. Only patients who lived in an area which had undergone heavy fighting between the Iraqi insurgents and the occupation forces and remained at the same area for at least 3 years were considered as potentially exposed patients. Those patients labelled as un-exposed lived in areas more than 10 KM from the regional fighting and also had the disease. All cases which were investigated and diagnosed were included. No patient refused to participate in the study, thus, no-response bias affects the final outcome of the work. To avoid any information bias, data was obtained from the electronic database by persons who were blinded to the research questions and outcome.

Normal specimens were also taken from non-malignant hysterectomy (without hyperplasia) for a control as part of standard RTPCR fold change detection.

All samples were formalin-fixed and paraffin embedded tissues. All cases were examined by two independent pathologists to confirm the diagnosis. FIGO system was used as the base for staging and grading of the tumor [15]. Since fixation can effect reliability of downstream RTPCR assays, protocols and timing for formalin fixation of all specimens were kept consistent.

### Sample size calculation

A sample size of 32 cases was calculated as the minimal requirement to conduct the present study. The study variable of interest was identified as dichotomous (proportion), and for these sample size calculations the required confidence level and width of the confidence interval was selected. The sample size was calculated using the equation: N = 4 * Zα2 * p (1 − p)/w2. Where Zα is the confidence level, W is the width of the confidence interval and P is the pre-study estimate of the proportion to be measured. N = 4 * 1.96 * 0.8(1 − 0.2)/0.2^2^ = 32 (required sample size in our study). The proportion of PTEN in endometrial carcinoma is about 80% with a selected confidence interval of ± 10 [16].

### RNA extraction and *PTEN* RTPCR gene expression

**Fig. 1:**
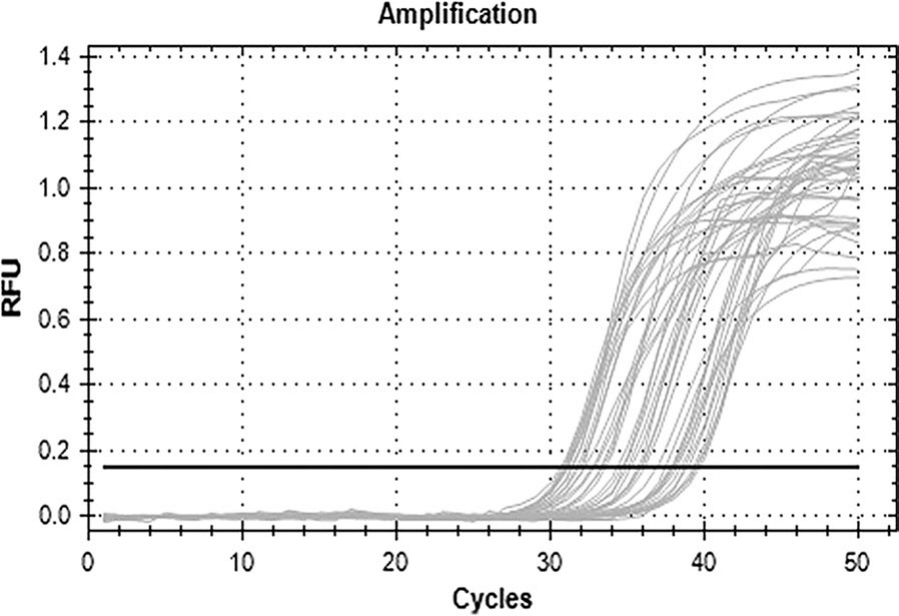
PTEN gene Ct value of malignant cases

PTEN gene expression was measured using RNA extracted from formalin-fixed and paraffin embedded tissue. Total RNA was extracted as previously described [17]. *PTEN* and *GAPDH* gene probes and primers were obtained from Bioneer company [South Korea]. *PTEN* gene expression was measured using quantitative real-time PCR. The *GAPDH* gene was used for normalization of malignant and benign samples of endometrial carcinoma according to the method described by Nolan et al. [17], Fig. 1 qRT-PCR curve. Both target and housekeeping gene data was analyzed using relative quantification gene expression level [fold change] as described by Levak [18]. For each test sample, to generate the relative expression levels, each of the normalized target values (CT values) were divided by the calibrator normalized target value, using the ΔCT method with the GAPDH reference gene.

#### Statistical analysis

The Qi Square test, exact test and Student’s t-test was used for statistical analysis using SPSS software programs version 23. P value at ≤ 0.05 was used as a level of significance.

## Results

### PTEN gene expression

The *PTEN gene* expression mean fold change in the non-exposed group was 0.0031 ± 0.0029 and 0.139 ± 0.185 in the exposed group, as shown in Table 1. The increase in the *PTEN gene* expression fold change in patients who lived in the (allegedly exposed) DU areas was significantly higher in comparison with the non-exposed group [P = 0.001]. No significant differences were observed in the age distribution between exposed [56.386 ± 7.84] and non-exposed [55.097 ± 8.0087] patients when the age of the subjects was considered [P = 0.45].

**Table 1:** PTEN fold change in relation to tumour characteristics in high risk [DU] exposed and non-exposed groups

PTEN fold change					
Group	Patient number	Mean PTEN	Standard deviation	Standard error	P value
Non-exposed	22	0.0031	0.0029	0.0006	0.001
Exposed	21	0.139	0.185	0.040	

Age distribution					
Group	Patient number	Mean age	Standard deviation	Standard error	P value
Non-exposed	22	56.38	7.84	1.18224	0.45
Exposed	21	55.09	8.0087	1.24076	

Grade differentiation in endometrial carcinoma					
Group	Grade			Total	P value
	Moderate	Poor	Well		
Non-exposed	8	1	13	22	0.286
Exposed	10	3	8	21	
Total	18	4	21	43	

Stage distribution in endometrial carcinoma					
Group	Stage				P value
	T1	T2	T3	Total	
Non-exposed	11	8	3	22	0.98
Exposed	10	8	3	21	
Total	21	16	6	43	

#### Tumour stage and grading in relation to gene expression

A total of 8 patients potentially exposed to depleted uranium were diagnosed with a well differentiated grade, 10 moderately differentiated and 3 with poorly differentiated tumors (Table 1). Thirteen patients of the non-exposed cohort were diagnosed with well differentiated tumour grade, 8 of which were moderately differentiated and 1 poorly differentiated. There were no significant differences in gene expression between the different groups when the grade of the tumors was considered for comparison [P = 0.286].

There were no significant differences in *PTEN gene* expression fold changes with regard to the stage of the tumor between the exposed and the non-exposed patients [P = 0.98]. In non-exposed subjects, 11 cases were of T1, 8 were T2 and 3 cases T3 stage. As for the exposed patients, 10 cases were of T1, 8 were T2 and 3 cases were of T3 stage [P = 0.98].

#### Involvement of cervix and ovary

Table 2 shows that the cervix was involved in five non-exposed cases and in four cases of the exposed patients. The study also found ovarian involvement in 2 of the non-exposed cases and 1 of the exposed group. There were no significant differences found between the two groups with respect to *PTEN* gene expression for both cervix [P = 0.532] and ovary [P = 0.518].

**Table 2:** Cervical and ovarian involvement of the studied groups

Cervical involvement in both high risk [DU] exposed and non-exposed patients				
Group	Cervical involvement			
	Free	Involved	Total	P value
Non-exposed	17	5	22	0.532
Exposed	17	4	21	
Total	34	9	43	

Ovarian involvements cross tabulation in high risk [DU] exposed and non-exposed groups				
Group	Ovarian involvement			P value
	Free	Involved	Total	
Non-exposed	20	2	22	0.518
Exposed	20	1	21	
Total	40	3	43	

## Discussion

DU has been increasingly used as a lethal component of munitions in military conflicts over the last two decades [19]. A level of around 320–800 tons of DU was estimated to be used in the first Gulf war in 1991. Nearly the same level was deployed in the 2nd Gulf war in 2003 [20, 21]. The carcinogenic effect of [DU] has been suggested using human samples [20–27].

This present study determined significant elevations in *PTEN* gene expression in endometrial cancer patients lived in areas allegedly polluted with DU when compared to patients in the unpolluted regions (Table 1). This work proves the severity of DNA damage linked to cancer inflicted on the Iraqi civilians by the use of DU-weaponry. This key finding of this study may provide an explanation for the high incidence of cancer in Iraq. This finding is further consolidated by our unpublished observations on the high frequency of sister chromatid exchanges among the Iraqi people who were living in allegedly [DU] exposed areas by comparing them with unexposed control [28]. DU may cause nonlethal mutations in critical genes associated with increased proliferation with mutant cells that lead to cancer. Future analysis of the mutational genetic landscape of patient tumours from DU-conflict areas will confirm this.

The present investigation showed that although there was a significant increase in fold change in *PTEN* gene expression in the high risk [DU] exposure group, other parameters including age, tumour stage and grading, cervix or ovarian involvement had no effect. *PTEN* gene expression was identified in all stages of tumor. A similar finding was reported by others who also reached the same conclusion [29–31]. This observation indicates that *PTEN* gene alteration is very important for tumour initiation and may synergise with other genetic alterations. Lastly, cervical involvement is an important parameter incorporated in the FIGO staging of endometrial carcinoma [15]. However, our work is also in agreement with other investigators who found no correlation between *PTEN* gene alteration and cervical involvement [32].

## Conclusion

In conclusion, further investigations at the molecular level will be required to clarify the mechanism by which DU may induce or accelerate network signaling pathways involving the *PTEN* gene linked to endometrial carcinogenesis. Strategies should also be considered and investigated for the therapeutic targeting of cancers with PTEN alterations to improve patient outlook.

## Limitation

Owing to the fact that the *PTEN gene* is a long gene with a total length of (128,336 bp), thus it is difficult to analyze it fully from formalin fixed paraffin embedded tissue. Accordingly, the present study investigated the whole gene through generating cDNA by isolating total RNA from formalin fixed paraffin embedded tissue.

## Data Availability

All the data used in the current study are not publicly available owing to institutional but may be requested on an individual basis from the corresponding author, Professor Akeel Yasseen.

## Abbreviations

PTEN: phosphatase and tensin homolog
DU: depleted uranium
RT-PCR: reverse transcription polymerase chain reaction
PI3 K: phosphatidylinositol-3-kinase
PIP3: phosphatidylinositol 3, 4, 5-triphosphate
